# Adoption of Precision Technologies by Brazilian Dairy Farms: The Farmer’s Perception

**DOI:** 10.3390/ani11123488

**Published:** 2021-12-07

**Authors:** Rebeca Silvi, Luiz Gustavo R. Pereira, Claudio Antônio V. Paiva, Thierry R. Tomich, Vanessa A. Teixeira, João Paulo Sacramento, Rafael E. P. Ferreira, Sandra G. Coelho, Fernanda S. Machado, Mariana M. Campos, João Ricardo. R. Dórea

**Affiliations:** 1 Department of Agricultural and Environmental Sciences, Universidade Estadual de Santa Cruz, Ilhéus 45662-900, BA, Brazil; rebecasilvy@yahoo.com.br; 2 Brazilian Agricultural Research Corporation—Embrapa Dairy Cattle, Juiz de Fora 36038-330, MG, Brazil; claudio.paiva@embrapa.br (C.A.V.P.); thierry.tomich@embrapa.br (T.R.T.); fernanda.machado@embrapa.br (F.S.M.); mariana.campos@embrapa.br (M.M.C.); 3 Department of Animal Science, Universidade Federal de Minas Gerais, Belo Horizonte 31270-901, MG, Brazil; vanessateixeiraamorim@gmail.com (V.A.T.); sandragesteiracoelho@gmail.com (S.G.C.); 4 Department of Biosystems Engineering, Universidade Federal de São João Del Rei, São João Del Rei 36301-160, MG, Brazil; jparvelos@yahoo.com.br; 5 Department of Animal and Dairy Sciences, University of Wisconsin, Madison, WI 53706, USA; referreira@wisc.edu

**Keywords:** cattle, sensor, livestock, smart farm, survey

## Abstract

**Simple Summary:**

Understanding the perception of dairy farmers regarding precision livestock technologies is crucial for creating strategic actions that will increase the rate of adoption and usage of such technologies. A survey study was applied to 378 dairy farms located in Brazil. The farmers were characterized based on technology usage, farmer profile, farm characteristics, and production indexes. The farms were classified into seven clusters: (1) top yield farms; (2) medium–high yield, medium-tech; (3) medium yield and top high-tech; (4) medium yield and medium-tech (5); young medium–low yield and low-tech; (6) elderly medium–low yield and low-tech; and (7) low-tech grazing. Our study helped to elucidate the farmer’s perception about precision technologies and to shed light on challenges that need to be addressed by scientific research and extension programs.

**Abstract:**

The use of precision farming technologies, such as milking robots, automated calf feeders, wearable sensors, and others, has significantly increased in dairy operations over the last few years. The growing interest in farming technologies to reduce labor, maximize productivity, and increase profitability is becoming noticeable in several countries, including Brazil. Information regarding technology adoption, perception, and effectiveness in dairy farms could shed light on challenges that need to be addressed by scientific research and extension programs. The objective of this study was to characterize Brazilian dairy farms based on technology usage. Factors such as willingness to invest in precision technologies, adoption of sensor systems, farmer profile, farm characteristics, and production indexes were investigated in 378 dairy farms located in Brazil. A survey with 22 questions was developed and distributed via Google Forms from July 2018 to July 2020. The farms were then classified into seven clusters: (1) top yield farms; (2) medium–high yield, medium-tech; (3) medium yield and top high-tech; (4) medium yield and medium-tech; (5) young medium–low yield and low-tech; (6) elderly medium–low yield and low-tech; and (7) low-tech grazing. The most frequent technologies adopted by producers were milk meters systems (31.7%), milking parlor smart gate (14.5%), sensor systems to detect mastitis (8.4%), cow activity meter (7.1%), and body temperature (7.9%). Based on a scale containing numerical values (1–5), producers indicated “available technical support” (mean; σ^2^) (4.55; 0.80) as the most important decision criterion involved in adopting technology, followed by “return on investment—ROI” (4.48; 0.80), “user-friendliness” (4.39; 0.88), “upfront investment cost” (4.36; 0.81), and “compatibility with farm management software” (4.2; 1.02). The most important factors precluding investment in precision dairy technologies were the need for investment in other sectors of the farm (36%), the uncertainty of ROI (24%), and lack of integration with other farm systems and software (11%). Farmers indicated that the most useful technologies were automatic milk meters systems (mean; σ^2^) (4.05; 1.66), sensor systems for mastitis detection (4.00; 1.57), automatic feeding systems (3.50; 2.05), cow activity meter (3.45; 1.95), and in-line milk analyzers (3.45; 1.95). Overall, the concerns related to data integration, ROI, and user-friendliness of technologies are similar to those of dairy farms located in other countries. Increasing available technical support for sensing technology can have a positive impact on technology adoption.

## 1. Introduction

There is a worldwide trend to optimize processes within a dairy farm, as the main challenge currently faced by producers is to balance high workloads with narrow profit margins. Increasingly demanding consumers, climate change, increased growth in population, and food quality and security are changing the decision-making processes used by dairy managers. Furthermore, the effectiveness of these processes will be key to increasing the profitability of dairy farming in a sustainable way [1,2].

Herd monitoring is one of the most complex challenges for dairy farms, especially on a large scale [3]. By taking into account the complexity of the activities performed in dairy farms, automatic monitoring can be an important tool for management decisions, which is generally based only on the experience and judgment of the producer [4]. The use of technology provides the means to improve management practices with an emphasis on farm efficiency of different herds’ sizes [1]. Usually, a sensor system consists of a device that measures physiological or behavioral parameters of an individual cow or herd, allowing the detection of deviations from normal animal conditions, which would require the producer’s attention [5].

Many options of precision dairy farming technologies are currently available; however, some farmers are simply unaware of them [6]. For example, devices and sensor systems can now be used to measure daily milk yield, cow activity, rumination, temperature, body weight (BW), body condition score (BCS), and many other events associated with animal health and welfare [7].

Several studies have investigated the adoption of technology by dairy farms around the world [3,8,9,10,11,12]. However, few studies provide information on which systems are widely used by dairy farms and which are rarely used. Moreover, the producers’ perspectives are not always taken into consideration when developing new technologies, and the reasons why farmers should invest in sensor systems may not yet be clear to them [13]. In addition, the adoption of certain technologies will require a large initial investment. As such, the dairy farmer needs to choose a system that will be easily integrated with other technologies in the short- and long-term future, have available technical support, and solve a critical problem in the farm. Unsuccessful investments have a strong impact on the farm’s financial health, and therefore, any investment must be considered with reasonable criteria [3].

The growing interest in technologies to reduce labor, maximize productivity, and increase profitability in a sustainable way is becoming noticeable in several countries, including Brazil. Information regarding technology adoption, perception, and effectiveness in dairy farms could shed light on challenges that need to be addressed by scientific research and extension programs. However, most of the current research has been developed in North America, Europe, and Oceania, and thus does not always apply to developing countries such as Brazil. In this context, we created and applied a survey to (i) characterize and cluster Brazilian dairy farms that adopt precision technologies, (ii) identify the main technologies used, and (iii) investigate the motivation behind investing in precision farming technologies.

## 2. Materials and Methods

### 2.1. The Data Collection

A 22-question survey was developed and distributed via the web, using Google Forms (Google Inc., Mountain View, CA, USA), from July 2018 to July 2020 (ethic committee process number CAAE 54354916.9.0000.5588). The producers’ contact information was obtained from databases of the Brazilian Association of Milk Producers (Abraleite), Holstein Association of Minas Gerais State, Brazilian Girolando Association, Milk Intelligence Center (CILeite/Embrapa), and MilkPoint. The survey was sent to potential respondents through uniform resource locator (URL) links distributed by dairy-related email list servers, as well as internet publications and print magazines volunteering to distribute the URL. In order to avoid sampling bias, the survey questionnaire was spread through all Brazilian geographic regions. Farmers who do not use milk machines were excluded from the survey, so it does not represent subsistence farms.

The survey consisted of 11 closed-ended and 11 open-ended questions (Supplemental Material). Respondents provided their email address and full name (questions 1, 2, and 3); the state and municipality where their farm was located (questions 4, 5); the property name (question 6); the respondent’s age and their role on the farm (questions 7 and 8); the number of employees (question 9); production system: confinement, semi-confinement, or grazing system (question 10); the participation of the producer and family members in the farm (question 11); current herd size (question 12); and daily milk production (question 13) and most used breed (question 14). Age, farmer role, current herd size, and daily milk yield were presented to respondents in categories. For the protection of the farmer’s privacy, farmer information was mapped to positive integers prior to any processing, with the reverse map being held in a different secure location.

Additionally, respondents were asked to identify the software herd management, the milking system type, and the precision dairy farming technologies available in the farm from a predetermined list (questions 15, 16, and 18). The technologies in the predetermined list were selected based on the work of [14] and technologies available in the Brazilian market. A scale containing numerical values (1–5) was used for the responses about the importance of predetermined factors in deciding to purchase precision dairy farming technologies (question 17). The respondents also used a scale (1–5) to classify the dairy farming technologies, based on usefulness, from the same list used in the technology adoption question (question 19). Producers were asked to mark with numbers from 1 to 3 the reasons for not investing in technologies from a predetermined list (question 20), and with numbers from 1 to 3, the problems they would like to solve with precision technologies (question 21).

### 2.2. Data Base and Analysis

A total of 448 farmers completed the survey. For sampling and data analysis, 70 farms were deleted because of errors or incomplete responses. The final data set consisted of 378 dairy farms distributed in 17 Brazilian states.

We performed a cluster analysis to group farms with a similar profile, and then we evaluated the technology adoption and usage within each cluster. We used the age of the farmer, number of staff members, production system, size of the herd, milk yield, and number of adopted technologies as input variables. The data analysis was performed in Python 3.6 using the Scikit-Learn module.

In order to be used in the clustering algorithm, all input variables had to be assigned numerical values, as some of them were categorical (e.g., production system), and some questions only specified a range of values (e.g., age and size of the herd). Each variable was converted to numerical values as follows: “age of the farmer” was converted to integer values between 0 and 4, with 0 corresponding to the first range (less than or equal to 30 years old), and 4 corresponding to the last range (greater than or equal to 60 years old); “number of staff members” kept the values as answered originally, as they were integers already; “production system” was assigned integer values between 0 and 2, with 0 corresponding to graze, 1 corresponding to semi-confined, and 2 corresponding to confined, following the increasing level of complexity of the systems; “size of the herd” was assigned the middle value for each range (the range “between 201 and 300 animals” was assigned value 250, for example), and the extreme ranges “less than or equal to 50 animals” and “more than or equal to 1001 animals” were assigned values 25 and 1250, respectively; “milk yield” was assigned the middle value for each range (the range “between 1001 and 2000 L per day” was assigned value 1500, for example), and the extreme ranges “less than or equal to 500 L per day” and “more than or equal to 30,001 L per day” were assigned values 250 and 40,000, respectively; “number of adopted technologies” kept the values as answered originally, as they were integers already.

All six variables were then normalized, meaning that, for each value, we subtracted the mean value for that variable and divided the result by the standard deviation of that variable:

$$X_{norm}=\frac{\left(X-\bar{X}\right)}{\sigma_{X}}$$

where $X_{norm}$ is the normalized value, X is the original value, $\bar{X}$ is the mean value of the variable, and $\sigma_{X}$ is the standard deviation of the variable.

Normalization was performed to ensure every variable was centered (mean equals 0) and scaled (standard deviation equal to 1). In this study, we wanted certain variables to have more impact on the final clusters, as we believed they were more relevant to the survey and questions proposed, so we then multiplied all values of each variable by a weight corresponding to that variable: “age of the farmer”, “number of staff members”, “production system”, and “size of the herd” were assigned weight 1; “milk yield” was assigned weight 2; and “number of adopted technologies” was assigned weight 3.

**Figure 1:**
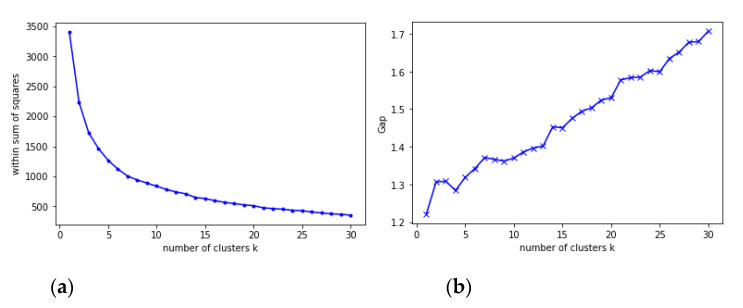
Results for (**a**) the within sum of squares and (**b**) the gap curve.

We then implemented the K-means clustering algorithm with the improvements proposed by [15], and we chose the number of clusters based on the gap statistic proposed by [16], analyzing up to 30 clusters and using the uniform distribution as the reference distribution. The results for the within sum of squares and the gap curve are shown in Figure 1.

Based on the results of the gap statistic method, we chose 7 as the number of clusters for the K-means algorithm, as this is where the gap curve reaches a local peak.

## 3. Results

The 378 farmers were distributed in seven clusters (Table 1). The first cluster included the top yield farms (1: Top Yield farms) and was characterized by an average of 31 staff members, ≥1001 dairy cows in the herd kept in a confined system (C), milk yield between 10,001 and 30,000 L/day, and high adoption of precision technologies (3.5 on average), with the age of the owners being between 41 and 50 years old. The second cluster (3: medium–high yield, medium-tech) included farms with an average of 15 staff members, 501 to 1000 dairy cows in the herd kept in a semi-confined system (SC), milk yield between 5001 and 10,000 L/day, and medium adoption of precision technologies (1.07 on average), with the age of the owner between 41 and 50 years old. The third cluster (3: medium yield and top high-tech) included farms with an average of 6 staff members, 201 to 300 dairy cows in the herd kept mainly in a confined system (C), milk yield between 2001 and 5000 L/day, and high adoption of precision technologies (6.67 on average), with an average age between 31 and 40 years old. The main characteristics of the fourth cluster (4: medium yield and medium high-tech) were an average of 5 staff members per farm, 101 to 200 dairy cows in the herd kept in a semi-confined system (SC), milk yield between 1001 and 2000 L/day, and medium adoption of precision technologies (2.74 on average), with the age of the owner varying between 31 and 40 years old. The fifth cluster (5: young, medium–low yield, and low-tech) was characterized by an average of 3 staff members per farm, 51 to 100 dairy cows in the herd kept in a confined system (C), milk yield between 501 and 1000 L/day, and low adoption of precision technologies (0.4 on average), with an average age between 31 and 40 years old. The sixth cluster (6: elderly, medium–low yield, and low-tech) presented an average of 3 staff members per farm, 101 to 200 dairy cows in the herd kept mainly in a confined system (C), milk yield between 501 and 1000 L/day, and low adoption of precision technologies (0.36 on average), with the age of the owner varying between 51 and 60 years old. The seventh cluster (7: low-tech grazing) was characterized by an average of 3 staff members per farm, 51 to 100 dairy cows in the herd kept in a pasture (G), milk yield below 500 L/day, and low adoption of precision technologies (0.41 on average), with the age of the owner varying between 41 and 50 years old.

The distribution of farms around the five Brazilian regions is shown in Table 2. The southwest region has the highest concentration of farms (44%), followed by the south (26%), northeast (16%), mid-west (13%), and north (1%) regions. We observed that most farms in the southeast and mid-west regions were included in clusters 5 (32% and 21%, respectively), 6 (26% and 27%, respectively), and 7 (14% and 21%, respectively); most farms with high technology adoption (cluster 3) were from the south region (42%). Based on the distributed survey, Top Yield farms (cluster 1) and high technology adoption (cluster 3) were not identified in the north and northeast regions.

The owners were considered as part of the staff in 62% of the farms participating in the survey (Table 3). In the current study, Girolando was the breed used in 48% of the farms, followed by Holstein (41%), Jersey (6%), and other breeds (4%). Holstein was the most used breed by farms in clusters 1 (62%), 2 (59%), 3 (92%), and 4 (72%); Girolando was the most used breed by farms in clusters 5 (49%), 6 (58%), and 7 (68%).

A total of 74% of the farms had a channeled milking system, and 26% of the farms adopted a bucket milking machine system (Table 4). The mid-level milk line was the most observed system in clusters 2 (56%), 5 (50%), 4 (45%), 6 (38%), and 1 (31%), and represents 40% of all respondent farms. Automatic milking systems (AMS) were observed only in clusters 3 and 4 (42% and 4%, respectively). We observed a wide adoption of the bucket milking machine system (62%) in cluster 7, characterized by pasture and low-tech farms.

The three main herd management software used by farmers are shown in Table 5. We observed that 29% of the farmers do not use management programs, and 23% use excel spreadsheets to manage the herd. Most of the farms that do not use software were located in clusters 6 and 7 (low tech clusters) and do not use management software (34% and 57%, respectively). Excel spreadsheets are the main farm management tool in clusters 5 and 4 (29% and 18%, respectively), and Ideagri software is the main program used by farms in clusters 1 and 2 (46% and 48%, respectively). The main software used by farmers in cluster 3 was Alpro/Delpro (33%). All farmers in clusters 1 and 3 (high tech clusters) adopt software as a herd management tool.

The three most important criteria used by farmers to purchase precision dairy farming technologies are presented in Table 6. The producers were asked to give a score from 1 to 5 to each pre-established criteria, and the “Availability of technical assistance” (mean; σ^2^) (4.55; 0.80) was the most important criterion, followed by “Cost-benefit ratio” (4.48; 0.80), “User-friendliness” (4.39; 0.88), “Investment cost” (4.36; 0.81) and “Management software compatibility” (4.2; 1,02). Based on clustering, farmers in clusters 1 (4.77; 0.33), 2 (4.70; 0.65), and 7 (4.39; 0.87) considered the “Cost-benefit ratio” as the main criterion for purchasing decision; the “Availability of technical assistance” was the most important criterion for producers in clusters 6 (4.57; 0.81), 5 (4.63; 0.54) and 3 (4.92; 0.08); and the “user-friendliness” was the most important criterion for producers in clusters 7 (4.39; 1.02) and 4 (4.43; 1.02).

Producers were asked to select the three most important reasons for not investing in precision technology (Table 7). “Preferring to invest in other areas” (36%, clusters 2, 3, 4, 5, 6 and 7), “Uncertainty about the ROI” (24%, clusters 2, 3, 4, 5, 6 and 7), and “Lack of integration with other farm systems and software” (11%, clusters 1, 2, 4, 6 and 7) are the most important factors pointed by Brazilian dairy farmers. Other reasons such as “There are other alternatives to improve daily management” (0.8%, cluster 1), “There is no technical assistance in the region” (4.3%, cluster 5), and “There is too much information/Know what to do with it” (0.5%, cluster 3), were also considered important for decision making.

Results regarding the use of precision dairy farming technologies are presented in Table 8. Automatic milk meters systems (31.7%), milking parlor smart-gate (14.5%), sensor systems for mastitis detection (electric conductivity) (8.4%), cow activity meter (collar activity, accelerometers, and pedometers) (7.1%), and body temperature sensor (7.9%) were the most adopted technologies (Table 8). Automatic milk meters systems were also the most common technologies found in farms in clusters 1, 2, 4, 5, and 6. Mastitis detector was the main technology in cluster 3, and milking parlor smart gate was the main technology in cluster 7. The least-used technologies were thermal infrared camera (0.8%), hormonal fertility sensors coupled to milk machine (0.8%), a sensor for detecting hoof health (0.8%), and GPS or animal location/position (0.8%).

The perceived usefulness of technologies is presented in Table 9. The most useful technologies pointed by farmers were milk meters systems (mean; σ^2^) (4.05; 1.66), sensor systems for mastitis detection (4.00; 1.57), automatic feeding systems (3.50; 2.05), cow activity meter (3.45; 1.95), and milk composition sensors (3.45; 1.95). Producers indicated GPS or animal location/position (2.85; 2.07), automatic body condition score (BCS) (2.91; 2.00), and thermal infrared camera (2.97; 2.04) to be the least useful. The milking parlor smart gate (clusters 1, 2, and 3), body temperature sensor (cluster 4), rumen activity sensor (clusters 1, 2, and 3), and automated calf feeder (cluster 6) were also pointed as useful technologies. 

The most important problems faced by producers are presented in Table 10. The main problems were “Mastitis” (39%), “Bovine Parasite Sadness (BPS)” (16%; clusters 4, 5, 6, and 7), and “labor” (15%; clusters 3, 5, 6, and 7). Other problems such as “Tick problems” (5.8%; clusters 4 and 5), “Health problems during peripartum” (1.6%; clusters 2 and 3), “High input costs” (0.7%; clusters 1), and “Residues management” (0.7%; clusters 1) were also considered important.

## 4. Discussion

The proposed clustering approach used in this article allowed us to (1) investigate the perception of dairy farmers grouped based on similar farm profiles regarding the adoption of precision livestock technologies, (2) compile the main technologies used in Brazilian dairy farms, and (3) the usefulness perception for each technology. An interactive report on a Power BI (Microsoft, Redmond, U.S.) platform is available at https://tinyurl.com/2epvdcuz (accessed on 20 May 2021), including data of all farms and clusters.

The level of milk yield, technology adoption, and herd size (L/day) decreased from clusters 1 to 7. Cluster 1 grouped farms with a high level of technology adoption (equipment and structure), herds with the largest number of high-producing cows, and the highest milk yield. On the other hand, cluster 7 grouped farms with low levels of technology adoption and small crossbred herds raised in grazing systems with low milk yield. In a review article [17], Stone used an adopter categories system proposed by Rogers [18], where five adopter categories were proposed: (1) innovators, (2) early adopters, (3) early majority, (4) late majority, and (5) laggards. They discussed how risk-averse a farmer is: at the two extremes, innovators are risk-takers, and laggards are extremely risk-averse. The farmers grouped in clusters 4 to 6 in our study are probably more risk-averse than those in clusters 1 to 3. Rogers explains that innovators adopt a new technology simply because it is new, which is a trait probably more evident in cluster 3 of the present study. They tend to be concerned with maintaining a reputation of being ahead of the curve [16]. The early majority, probably represented in our study by clusters 1 and 2, make adoption decisions based on utility and practical benefit, as opposed to reputation or the newness factor. Alternatively, the laggards (clusters 4, 5, and 6) are slower to adopt new technologies and only do so when they are forced to by some external force, for example, the mandatory criteria used by the Ministry of Agriculture, Livestock and Food Supply regarding the requirements for the production, storage, conservation, and transport of raw milk [19].

The most productive dairy farms were concentrated in the south and southeast regions of Brazil [17], and this concentration was also observed in our study. Minas Gerais state is included in this region and has the largest dairy herd (3.1 million head) in the country and is responsible for 27.1% of all milk produced in Brazil [20]. The lowest percentage of owners working in the property (50%) was observed in cluster 3, where farms with the highest level of automation were grouped. In contrast, the highest percentage (75%) was observed in cluster 5, characterized by low adoption of technology. The Holstein is the main explored breed in farms with higher productivity and adoption of precision technology. In 68% of the farms with a grazing system, there is a preference for Girolando cows, which is probably due to the better adaptability of this crossbreed to grazing tropical pasture.

According to the clustering, farms with modern barns and specialized equipment usually had automatic milking systems (AMS). In these farms, the herd management software used was compatible with the AMS. AMS is a relatively recent technology in Brazil and is still not widely used. It represents a higher capital investment compared to conventional systems [21] and requires owners to adapt to a new management system [22]. 

Low-Tech Grazing farms (cluster 7) and medium-low yield and low-tech farms (Cluster 6) reported the highest level of no use of herd management software (57 and 34%, respectively). Excel spreadsheets were more common in farms with medium yield and medium-tech (Cluster 4) and medium-low yield and low-tech (Cluster 5), and 100% of top yield farms (medium-high yield, medium-tech) and medium yield and top high-tech reported the adoption of some type of herd management software. According to [23], herd management programs can support producers in changing from “curative” to “preventive” management decisions, which becomes more important as the herd size increases. There was a trend in farmers with larger herds to adopt more precision technologies when compared to farmers with smaller herds. Moreover, the adoption of technologies is still low, especially in farms with less than 500 animals in the herd. The same scenario was observed by [12] in Australian farms.

Our results regarding the criteria considered for purchasing precision dairy farming technologies are in agreement with [3], in a survey performed on American dairy farms. The owners indicated the cost:benefit ratio, total investment cost, user-friendliness, and the availability of technical assistance as determining factors for the adoption of technologies. All factors mentioned in the question can be considered important to evaluate the decision to adopt precision technologies since all criteria had a score above 4 when the maximum selectable value was 5. Clusters 5, 6, and 7 (low adoption of technology) attributed lower scores compared to clusters 1 and 3, which have a higher level of technology adoption.

The most important reasons for not investing in precision technology corroborate with the results observed by [11] in the Netherlands. The reasons for not investing in new technologies pointed by Dutch farmers were “Prefer to invest money in other things for the farm”, “uncertainty about the ROI” and “Lack of integration with other farm systems and software”.

“Automatic milk meters systems” was the technology most used for clusters 1, 2, 3, 4, 5, and 6, followed by “Milking parlor smart gate” for clusters 1, 2, 4, 5, and 6. In contrast, the sensors used to detect mastitis and activity were the technologies most used in North American [3] and Dutch [11] farms. Such technologies are more world widely used, especially when compared to technologies that generate variables less useful for the producers [3], such as “infrared thermal camera” technology, less used by farmers in our study. The high level of adoption of “automatic milk meters systems” by all clusters can be explained by the usefulness of this technology, graded with the highest score (Table 9). The variables retrieved from this technology can be used, for example, to predict cow disorders in dairy herds [24] and to identify the energy balance status of a lactating cow [25]. The cow activity meter is the oldest parameter used in monitoring dairy cattle and was characterized for the first time as precision technologies by [23], and can be used as a tool for estrus detection and calving prediction [24,25,26]. Although, for 80% of cluster 3 farmers who used the activity meter, those sensors were only the fourth most used technology and were graded as the fourth-highest score of usefully. In contrast, infrared thermal camera, hormonal fertility sensors, and a sensor for detecting hoof health are relatively new technologies on the market and therefore more rarely commercialized [3] and used by farmers of North America, Europe, and Brazil. 

We observed differences in the perceived usefulness of technologies by producers according to their cluster in Table 9. In general, the producers in cluster 7, with low levels of technology adoption, also evaluate technologies as being less useful. The degree of compatibility of the technology can explain the result. According to [26], compatibility is the degree to which a technology is considered consistent with the profile of the property or a producing region. For example, the scale of the utility of equipment and sensors coupled to milking was the lowest in cluster 7, which has the bucket milking machine as the main system. Usually, the technologies considered most useful by farmers were also the ones that producers are the most familiar with it. Moreover, some technologies require qualified labor and changes in farm management activities, which can lead owners to evaluate the equipment negatively [3].

Regarding the three most important problems faced by farmers in the survey, mastitis, the main one, can be detected through sensors that measure the electrical conductivity (EC) and the CCS of milk [27]. The authors of [28] reported that it is possible to determine the occurrence of mastitis with an accuracy of 77% through gas sensors capable of evaluating samples of raw milk in terms of acidity, composition, CCS, and electrical conductivity. Bovine parasite sadness (tick fever), the second most important problem according to farmers, can be detected by precision technologies [29]. Labor problems (third most important) can also be solved or minimized through the adoption of precision technologies. For example, precision technologies were adopted by Australian farmers to automate the milking room cleaning systems [12].

The dairy sector is facing a worldwide trend of small farms disappearing as large farms grow even larger, and this trend is shaping the precision technology market. For example, the number of dairy farmers in the United States [26] decreased by 40% between 2010 and 2020 (from about 53,100 to 31,600), while during that same interval, milk production increased by 15% (from 87.5 to 101.2 billion liters per year), as the total number of cattle remained nearly constant (from 9.1 to 9.4 million). This suggests an intensification of milk production processes across the country, possibly accompanied by an increase in technology usage. Conversely, in Brazil [17], the number of dairy farmers decreased by 13% between 2006 and 2017 (from 1.35 to 1.17 million), while milk production increased by 47% (from 20.5 to 30.2 billion liters per year), and the total number of cattle decreased by 9% (from 12.7 to 11.5 million). However, of the 1.17 million dairy farmers in Brazil, few have adopted precision technology, such as the farmers in clusters 1 to 3. Additionally, milk production is very concentrated in large dairy farms: only 2% of Brazilian dairy farmers produce more than 500 L of milk a day, and less than 0.5% produce more than 1000 L a day. Nevertheless, the number of farmers producing more than 500 L of milk a day increased by 165%, while the number of those producing less than 500 L a day decreased by 14% between 2006 and 2017. Such fluctuation elucidates the potential for the adoption of precision technologies in Brazil, as high-producing and efficient farms can greatly benefit from adopting technology.

Interestingly, farms that use grazing systems, grouped in cluster 7, showed the lowest level of technology adoption. However, some pasture-based farms were included in clusters characterized as with medium and high technology adoption. This low adoption may be related to the few technologies designed specifically to increase pasture utilization [30]. Some intensive pasture-based farms fall within the Top 100 milk producers in Brazil, showing the strength of grazing systems in tropical environments. Thus, the development and adaptation of useful precision technologies for grazing systems can help leverage their profitability.

## 5. Conclusions

The adoption of precision technologies by Brazilian farms is still considered low, providing manufacturers, researchers, and educators an opportunity to explore the sector. Technologies for monitoring milking performance and udder health were the most widely used. The main reasons for not investing in precision technologies were related to economic issues and the farmer’s lack of knowledge about the technologies or the importance of the parameters monitored by them. However, the study points to higher productivity in farms with higher levels of technology adoption. Overall, the concerns related to data integration, ROI, and user-friendliness are similar to other dairy farms located in other countries. Increasing the availability of technical support to users can have a positive impact on precision technology adoption. 

## Figures and Tables

**Table 1:** Farms’ average traits based on variables used in the cluster analyses.

Cluster.	Farms	Farmers Age			Staff		Production System			Total Animals (*n*)			Milk			Technologies	
	*n*	Years	Score	σ^2^	*n*	σ^2^	Sys *	Score	σ^2^	Herd Size	Score	σ^2^	Yield (kg)	Score	σ^2^	*n*	σ^2^
1	13	41 a 50	1.85	1.21	31	177	C*^i^*	1.69	0.37	≥1001	5.92	0.07	10,001 to 30,000	5.15	0.13	3.15	3.21
2	27	41 a 50	1.63	1.20	15	54	C	1.63	0.31	501 to 1000	4.56	1.28	5001 to 10,000	3.93	0.29	1.07	1.18
3	12	31 a 40	1.42	1.24	6	24	C	1.83	0.31	201 to 300	2.75	1.85	2001 to 5000	2.83	1.31	6.67	2.22
4	50	31 a 40	0.98	1.18	5	14	S^ii^-C	1.46	0.29	101 to 200	2.38	1.64	1001 to 2000	2.26	1.23	2.74	0.51
5	112	31 a 40	0.53	0.25	3	6	S-C	1.17	0.14	51 to 100	1.46	1.39	501 to 1000	1.23	1.32	0.40	0.24
6	108	51 a 60	2.70	0.58	3	5	S-C	1.18	0.14	101 to 200	1.60	1.28	501 to 1000	1.16	1.17	0.36	0.29
7	56	41 a 50	1.91	1.30	3	33	G^iii^	0.00	0.00	51 to 100	1.07	1.99	≤500	0.41	0.56	0.29	0.28

(1) Top Yield farms; (2) Medium–High Yield, Medium-Tech; (3) Medium Yield and Top High-Tech; (4) Medium Yield and Medium-Tech (5) Young, Medium–Low Yield and Low-tech; (6) Elderly, Medium-Low Yield and Low-Tech; and (7) Low-Tech Grazing. * Production System: (*i*) confinement system (free-stall, tie-stall and compost barn), (*ii*) Semi-confinement, and (*iii*) grazing system.

**Table 2:** Regional localization of farms clusters in Brazilian regions.

Cluster	Farms	Southest		South		Northeast		Mid West		North	
	*n*	*n*	%	*n*	%	*n*	%	*n*	%	*n*	%
1	13	6	46	4	31	0	0	3	23	0	0
2	27	14	52	5	19	5	1	3	11	0	0
3	12	4	33	5	42	0	0	3	25	0	0
4	50	20	40	23	46	2	1	5	10	0	0
5	112	53	47	38	34	19	21	11	10	0	0
6	108	44	41	17	16	21	23	14	13	2	2
7	56	24	43	5	9	12	7	12	21	2	4
All farms	378	165	44	97	26	59	16	51	13	4	1

(1) Top Yield Farms; (2) Medium–High Yield, Medium-Tech; (3) Medium Yield and Top High-Tech; (4) Medium Yield and Medium-Tech; (5) Young, Medium–Low Yield and Low-Tech; (6) Elderly, Medium–Low Yield and Low-Tech; and (7) Low-Tech Grazing.

**Table 3:** Percentege of farms that the owner is part of staff and breed used by farms clusters.

Cluster	Farms	Owner Is Part of Farm Staff		Breed							
				Girolando		Holstein		Jersey		Others	
	*n*	*n*	%	*n*	%	*n*	%	*n*	%	*n*	%
1	13	9	69	5	38	8	62	0	0	0	0
2	27	14	52	9	33	16	59	0	0	2	7
3	12	6	50	1	8	11	92	0	0	0	0
4	50	33	66	10	20	36	72	4	2	0	0
5	112	84	75	55	49	45	40	9	10	3	3
6	108	57	53	63	58	29	27	7	8	8	7
7	56	35	63	38	68	11	20	3	2	4	7
All farms	378	235	62	181	48	156	41	24	6	17	4

(1) Top Yield Farms; (2) Medium–High Yield, Medium-Tech; (3) Medium Yield and Top High-Tech; (4) Medium Yield and Medium-Tech; (5) Young, Medium–Low Yield and Low-Tech; (6) Elderly, Medium–Low Yield and Low-Tech; and (7) Low-Tech Grazing.

**Table 4:** Milking machine system used by farms cluster.

Cluster	Farms	Low-Level Milkline		Midlevel Milkline		High-Level Milkline		Side by Side		Rotatory		AMS		Bucket M. Machine	
	*n*	*n*	%	*n*	%	*n*	%	*n*	%	*n*	%	*n*	%	*n*	%
1	13	3	23	4	31	0	0	5	38	1	8	0	0	0	0
2	27	11	41	15	56	0	0	0	0	0	0	0	0	1	4
3	12	4	33	2	17	0	0	1	8	0	0	5	42	0	0
4	49	18	37	22	45	2	4	4	8	0	0	2	4	1	2
5	111	12	11	56	50	8	7	7	6	0	0	0	0	28	25
6	105	15	14	38	36	10	10	9	9	0	0	0	0	33	31
7	53	1	2	11	21	7	13	1	2	0	0	0	0	33	62
All farms	370	64	17	148	40	27	7,3	27	7	1	0	7	2	96	26

(1) Top Yield Farms; (2) Medium–High Yield, Medium-Tech; (3) Medium Yield and Top High-Tech; (4) Medium Yield and Medium-Tech; (5) Young, Medium–Low Yield and Low-tech; (6) Elderly, Medium–Low Yield and Low–Tech; and (7) Low-Tech Grazing.

**Table 5:** Top three herd management software used by farmers.

Cluster	Farms	Management Softwares										
	*n*	First	*n*	%	Second	*n*	%	Third	*n*	%	Others	%
1	13	Ideagri	6	46	DairyPlan	3	23	Alpro/Delpro	2	15	2	15
2	27	Ideagri	13	48	Alpro/Delpro	3	11	Excel	3	11	8	30
3	12	Alpro/Delpro	4	33	DairyPlan	3	25	ABS Monitor	1	8	4	33
4	50	Excel	9	18	Not use	9	18	Ideagri	7	14	24	49
5	112	Excel	36	32	Not use	33	29	Ideagri	12	11	31	28
6	108	Not use	36	34	Excel	27	26	Prodap	16	15	26	25
7	56	Not use	30	57	Excel	13	25	Prodap	5	9	5	9
All farms	378	Not use	110	29	Excel	88	23	Ideagri	50	13	130	34

(1) Top Yield Farms; (2) Medium–High Yield, Medium-Tech; (3) Medium Yield and Top High-Tech; (4) Medium Yield and Medium-Tech; (5) Young, Medium–Low Yield and Low-Tech; (6) Elderly, Medium–Low Yield and Low-Tech; and (7) Low-Tech Grazing.

**Table 6:** Decision key criteria to purchase a precision technology.

Cluster	Farms	Availability of Technical Assistance		Cost-Benefit Ratio		User-Friendliness		Investment Cost		Management Software Compatibility	
	*n*	Avarage *	σ^2^	Avarage *	σ^2^	Avarage*	σ^2^	Avarage *	σ^2^	Avarage *	σ^2^
1	13	4.54	0.40	4.77	0.33	4.38	0.54	4.23	0.79	4.69	0.37
2	27	4.67	0.67	4.70	0.65	4.44	0.84	4.48	0.69	4.52	0.84
3	12	4.92	0.08	4.42	0.41	4.58	0.24	4.58	0.41	4.50	0.75
4	50	4.40	1.28	4.40	1.04	4.43	1.02	4.31	1.03	4.20	1.24
5	112	4.63	0.54	4.48	0.75	4.34	0.94	4.41	0.75	4.24	0.93
6	108	4.57	0.81	4.48	0.82	4.40	0.80	4.32	0.77	4.30	0.98
7	56	4.35	1.08	4.39	0.87	4.39	1.02	4.30	0.93	4.07	1.25
All farms	378	4.55	0.80	4.48	0.80	4.39	0.88	4.36	0.81	4.27	1.02

(1) Top Yield Farms; (2) Medium Yield and Top High-Tech; (3) Medium–High Yield, Medium-Tech; (4) Medium Yield and Medium-Tech; (5) Young, Medium–Low Yield and Low-Tech; (6) Elderly, Medium–Low Yield and Low-Tech; and (7) Low-Tech Grazing. * Values calculated by assigning the following values to response categories: not important = 1; of little impotance = 2; moderately impotance = 3; important = 4; very important = 5.

**Table 7:** Top three reasons to not invest in precision technologies.

Cluster	Farms	Reasons								
	*n*	First	*n*	%	Second	*n*	%	Third	*n*	%
**1**	10	Uncertainty about the ROI	3	30	There are other alternatives to improve daily management	3	30	Lack of integration with other farm systems and software	3	30
**2**	27	Prefer to invest in other areas	13	48	Uncertainty about the profitability of the investment	8	30	Lack of integration with other farm systems and software	3	11
**3**	12	Prefer to invest in other areas	6	50	Uncertainty about the ROI	4	33	There is too much information/Know what to do with it	2	17
**4**	49	Prefer to invest in other areas	18	37	Uncertainty about the ROI	17	35	Lack of integration with other farm systems and software	11	22
**5**	112	Prefer to invest in other areas	43	38	Uncertainty about the ROI	25	22	There is no technical support in the region	16	14
**6**	105	Prefer to invest in other areas	29	28	Uncertainty about the ROI	21	20	Lack of integration with other farm systems and software	14	13
**7**	53	Prefer to invest in other areas	22	42	Uncertainty about the ROI	14	26	Lack of integration with other farm systems and software	10	19
All farms	368	Prefer to invest in other areas	131	36	Uncertainty about the ROI	89	24	Lack of integration with other farm systems and software	41	11

(1) Top Yield Farms; (2) Medium–High Yield, Medium-Tech; (3) Medium Yield and Top High-Tech; (4) Medium Yield and Medium-Tech; (5) Young, Medium–Low Yield and Low-Tech; (6) Elderly, Medium–Low Yield and Low-Tech; and (7) Low-Tech Grazing.

**Table 8:** Technologies used by farmers.

	Cluster (*n* and %)															
	1		2		3		4		5		6		7		Total	
Technology	13	%	27	%	12	%	50	%	112	%	108	%	56	%	378	%
Automatic milk meters systems	10	*77*	14	*52*	10	*83*	36	72	28	25	19	*18*	3	*5*	120	*32*
Milking parlor smart gate	6	*46*	3	*11*	8	*67*	15	30	7	6	9	*8*	7	*13*	55	*15*
Sensor systems for mastitis detection	5	*38*	1	*4*	12	*100*	12	24	0	0	2	*2*	0	*0*	32	*8*
Cow activity meter	4	*31*	1	*4*	10	*83*	11	22	0	0	0	*0*	1	*2*	27	*7*
Body temperature sensor	0	*0*	2	*7*	4	*33*	12	24	4	4	2	*2*	2	*4*	26	*7*
Automated feeding system	1	*8*	0	*0*	4	*33*	13	26	3	3	3	*3*	0	*0*	24	*6*
Rumen activity sensor	3	*23*	1	*4*	6	*50*	10	20	0	0	1	*1*	0	*0*	21	*6*
Automatic body weighing platform	2	*15*	3	*11*	1	*8*	7	14	3	3	2	*2*	2	*4*	20	*5*
Automated calf feeder	3	*23*	1	*4*	6	*50*	5	10	0	0	1	*1*	0	*0*	16	*4*
NIRs	2	*15*	1	*4*	3	*25*	5	10	0	0	0	*0*	0	*0*	11	*3*
Eletronic feed and drink bins	0	*0*	0	*0*	5	*42*	4	8	0	0	0	*0*	0	*0*	9	*2*
In-line milk analyzers	1	*8*	0	*0*	4	*33*	1	2	0	0	0	*0*	0	*0*	6	*2*
Automated BCS sensor	1	*8*	0	*0*	2	*17*	1	2	0	0	0	*0*	0	*0*	4	*1*
Respiration rate sensor	0	*0*	1	*4*	2	*17*	1	2	0	0	0	*0*	0	*0*	4	*1*
Ruminal pH sensor	0	*0*	0	*0*	1	*8*	0	0	1	1	1	*1*	1	*2*	4	*1*
GPS or animal location/position	0	*0*	0	*0*	1	*8*	1	2	0	0	0	*0*	1	*2*	3	*1*
Sensors for detecting hoof health	1	*8*	1	*4*	1	*8*	0	0	0	0	0	*0*	0	*0*	3	*1*
Hormonal fertility sensors coupled to mechanical milking	1	*8*	0	*0*	1	*8*	1	2	0	0	0	*0*	0	*0*	3	*1*
Infrared thermal camera	1	*8*	0	*0*	0	*0*	2	4	0	0	0	*0*	0	*0*	3	*1*

(1) Top Yield Farms; (2) Medium–High Yield, Medium-Tech; (3) Medium Yield and Top High-Tech; (4) Medium Yield and Medium-Tech; (5) Young, Medium–Low Yield and Low-Tech; (6) Elderly, Medium–Low Yield and Low-Tech; and (7) Low-Tech Grazing.

**Table 9:** Farmers’ perception about the usefulness of different precision technologies.

	Cluster															
Technology	1		2		3		4		5		6			7	Total	
	Score *	σ^2^ **	Score	σ^2^	Score	σ^2^	Score	σ^2^	Score	σ^2^	Score	σ^2^	Score	σ^2^	Score	σ^2^
Automatic milk meters systems	4.15	*1.05*	4.41	0.98	4.33	*0.72*	4.51	0.82	4.06	*1.67*	3.98	1.70	3.48	*2.44*	4.05	*1.66*
Milking parlor smart gate	4.17	*1.81*	3.74	1.53	4.00	*1.00*	3.40	1.74	3.18	*1.91*	3.51	1.50	3.17	*2.37*	3.40	*1.84*
Sensor systems for mastitis detection	3.67	*1.56*	3.73	1.35	4.25	*1.02*	4.04	1.58	4.03	*1.50*	4.18	1.54	3.73	*1.77*	4.00	*1.57*
Cow activity	3.69	*1.60*	3.38	1.93	4.17	*1.47*	3.74	1.72	3.40	*1.84*	3.49	1.89	3.04	*2.34*	3.45	*1.95*
Body temperature sensor	3.46	*2.56*	3.12	1.95	3.58	*1.58*	3.65	1.35	3.18	*1.86*	3.19	1.95	2.98	*2.29*	3.24	*1.94*
Automated feeding system	2.83	*2.64*	2.81	2.23	3.67	*1.06*	3.73	1.53	3.49	*1.94*	3.86	1.88	3.08	*2.22*	3.50	*2.05*
Rumen activity sensor	3.77	*1.72*	3.19	2.00	3.75	*2.19*	3.52	1.77	3.04	*1.92*	3.04	2.00	2.78	*2.29*	3.13	*2.05*
Automatic body weighing platform	2.92	*2.58*	2.85	1.59	2.75	*1.19*	3.21	1.36	3.15	*1.96*	3.29	1.85	3.21	*2.44*	3.16	*2.05*
Automated calf feeder	3.25	*2.35*	2.81	1.85	3.50	*1.58*	3.52	1.33	3.07	*1.72*	3.29	1.99	3.23	*2.14*	3.22	*1.87*
NIRs	2.58	*2.41*	2.76	2.18	3.17	*1.97*	3.63	1.54	3.09	*2.10*	3.13	1.94	2.61	*2.32*	3.06	*2.12*
Eletronic feed and drink bins	3.08	*2.74*	2.92	2.15	3.25	*1.52*	3.30	1.57	3.40	*1.96*	3.48	1.93	3.10	*2.51*	3.31	*2.04*
In-line milk analyzers	3.58	*1.41*	2.77	1.87	3.25	*1.52*	3.51	1.65	3.50	*2.00*	3.62	1.86	3.33	*2.22*	3.45	*1.95*
Automated BCS sensor	2.08	*1.58*	2.38	1.70	2.92	*1.41*	3.31	1.55	2.93	*2.03*	3.01	1.97	2.77	*2.33*	2.91	*2.00*
Respiration rate sensor	2.77	*2.64*	2.88	1.95	3.33	*1.72*	3.35	1.71	3.08	*1.89*	2.96	1.68	2.71	*2.32*	3.01	*1.93*
Ruminal pH sensor	2.08	*2.08*	2.73	1.74	3.33	*1.22*	3.47	1.44	3.26	*1.87*	3.05	1.85	2.94	*2.09*	3.11	*1.89*
GPS or animal location/position	2.25	*2.02*	2.46	1.63	2.83	*1.97*	3.04	1.78	2.85	*2.09*	2.90	2.17	2.90	*2.13*	2.85	*2.07*
Sensor for detecting hoof health	2.17	*1.64*	3.00	2.46	3.50	*1.75*	3.53	1.65	3.32	*1.74*	3.25	1.93	2.96	*2.15*	3.22	*1.96*
Hormonal fertility sensors	3.58	*1.41*	2.77	1.87	3.25	*1.52*	3.51	1.65	3.50	*2.00*	3.62	1.86	3.33	*2.22*	3.45	*1.95*
Infrared thermal camera	2.25	*2.35*	2.62	1.85	3.25	*1.69*	3.33	1.48	3.08	*2.02*	2.92	1.99	2.79	*2.36*	2.97	*2.04*
Number of technologies/farm	3.07	*2.01*	3.02	1.83	3.48	*1.48*	3.54	1.54	3.30	*1.90*	3.36	1.87	3.06	*2.26*	3.29	1.94

(1) Top Yield Farms; (2) Medium–High Yield, Medium-Tech; (3) Medium Yield and Top High-Tech; (4) Medium Yield and Medium-Tech; (5) Young, Medium–Low Yield and Low-Tech; (6) Elderly, Medium–Low Yield and Low-Tech; and (7) Low-Tech Grazing. * Values calculated by assigning the following values to response categories: not useful = 1; of little usefulness = 2; moderately useful = 3; useful = 4; very useful = 5; ** variance.

**Table 10:** Top three problems faced by farmers.

Cluster	Farms	Reasons								
	*n*	First	*n*	%	Second	*n*	%	Third	*n*	%
1	13	Mastitis/BPS *	3	23	High input costs	3	23	Manure management	3	23
2	27	Mastitis	8	30	Mastitis	5	19	Peripartum problems *	4	15
3	12	Mastitis	4	33	Peripartum problems **	2	17	Labor	2	17
4	50	Mastitis	25	50	BPS	13	26	Tick	9	18
5	112	Mastitis	45	40	BPS	15	13	Labor/Tick	13	12
6	108	Mastitis	42	39	BPS	18	17	Labor	21	19
7	56	Mastitis	21	38	BPS	10	18	Labor	8	14
All farms	378	Mastitis	148	39	BPS	59	16	Labor	56	15

(1) Top Yield farms; (2) Medium–High Yield, Medium-Tech; (3) Medium Yield and Top High-Tech; (4) Medium Yield and Medium-Tech; (5) Young, Medium–Low Yield and Low-Tech; (6) Elderly, Medium–Low Yield and Low-Tech; and (7) Low-Tech Grazing * Bovine Parasite Sadness (or tick fever). ** Including metabolic and reproductive problems.

## Data Availability

The data presented in this study are available at https://tinyurl.com/2epvdcuz (accessed on 20 May 2021).

## Supplementary Materials

The survey is available online at https://tinyurl.com/nn4ea79k and PBI 511 dashboard at https://tinyurl.com/2epvdcuz (accessed on 20 May 2021).
